# Biological Synthesis of Monodisperse Uniform-Size Silver Nanoparticles (AgNPs) by Fungal Cell-Free Extracts at Elevated Temperature and pH

**DOI:** 10.3390/jof8050439

**Published:** 2022-04-23

**Authors:** Mariana Fuinhas Alves, Patrick G. Murray

**Affiliations:** Shannon Applied Biotechnology Centre, Department of Applied Science, Faculty of Applied Sciences and Technology, Moylish Campus, Technological University of the Shannon, Midlands Midwest, Moylish, V94 EC5T Limerick, Ireland; patrick.murray@lit.ie

**Keywords:** biosynthesis, fungus, nanotechnology, reaction optimisation, silver nanoparticles

## Abstract

Fungi’s ability to convert organic materials into bioactive products offers environmentally friendly solutions for diverse industries. In the nanotechnology field, fungi metabolites have been explored for green nanoparticle synthesis. Silver nanoparticle (AgNP) research has grown rapidly over recent years mainly due to the enhanced optical, antimicrobial and anticancer properties of AgNPs, which make them extremely useful in the biomedicine and biotechnology field. However, the biological synthesis mechanism is still not fully established. Therefore, this study aimed to evaluate the combined effect of time, temperature and pH variation in AgNP synthesis using three different fungi phyla (Ascomycota, Basidiomycota and Zygomycota) represented by six different fungi species: *Cladophialophora bantiana* (*C*. *bantiana*), *Penicillium antarcticum* (*P. antarcticum*), *Trametes versicolor* (*T. versicolor*), *Trichoderma martiale* (*T. martiale*), *Umbelopsis isabellina* (*U. isabellina*) and *Bjerkandera adusta* (*B. adusta*). Ultraviolet–visible (UV-Vis) spectrophotometry and transmission electron microscopy (TEM) results demonstrated the synthesis of AgNPs of different sizes (3 to 17 nm) and dispersity percentages (25 to 95%, within the same size range) using fungi extracts by changing physicochemical reaction parameters. It was observed that higher temperatures (90 °C) associated with basic pH (9 and 12) favoured the synthesis of monodisperse small AgNPs. Previous studies demonstrated enhanced antibacterial and anticancer properties correlated with smaller nanoparticle sizes. Therefore, the biologically synthesised AgNPs shown in this study have potential as sustainable substitutes for chemically made antibacterial and anticancer products. It was also shown that not all fungi species (*B. adusta*) secrete metabolites capable of reducing silver nitrate (AgNO_3_) precursors into AgNPs, demonstrating the importance of fungal screening studies.

## 1. Introduction

The conversion of organic materials into bioactive products by fungi offers sustainable solutions, especially as substitutes for toxic chemicals, which is extremely important for a bio-based circular economy [1,2]. Metabolites secreted by fungi in cell-free extracts are bioactive compounds with various applications. Hence, there is an increase in fungal biotechnology research, particularly focusing on fungal growth optimisation and potential applications of the metabolites secreted by fungi [1,2]. One application of fungi that has been explored is in the nanotechnology field in green bio-based nanoparticle synthesis.

The resistance to toxic heavy metals displayed by microorganisms, such as the bacteria *Pseudomonas aeruginosa* and the fungus *Aspergillus niger*, both used in chemical detoxification, inspired the development of greener routes to synthesise nanomaterials [3,4]. Nowadays, the use of hazardous chemicals and toxic reducing and stabilising agents in nanoparticle synthesis are gradually being replaced with more sustainable, safe and cost-effective synthesis routes [3,5]. As a result, these agents are being substituted by biological agents such as viruses, bacteria, fungi, yeasts, algae and plants or bio-extracted compounds such as proteins/peptides, carbohydrates and vitamins [6,7,8].

The benefits of eco-friendly biological synthesis include enormous biodiversity, environmental compatibility, scalability, energy-efficient methodologies, low or reduced production cost, and less toxic or nontoxic end-products [8,9,10,11,12,13,14]. However, biological methods mainly produce nanoparticle solutions with heterogeneous morphologies, and the synthesis mechanism is still not fully established [3,15,16,17].

According to the International Organization for Standardization (ISO), a nanoparticle is a “nano-object, [discrete piece of material with one, two or three external dimensions in the nanoscale (length range approximately from 1 nm to 100 nm)], with all external dimensions in the nanoscale where the lengths of the longest and the shortest axes of the nano-object do not differ significantly” [18]. The physicochemical properties of nanoparticles are determined by their morphology. In other words, their properties are size- and shape-dependent. It has been previously demonstrated that nanoparticle size can be tuned by changing reaction parameters such as salt precursor concentration, stabilising agents, time, temperature and pH [19,20,21]. However, in the biological green myco-synthesis field, the metabolites involved in reducing and stabilising the nanoparticles can be distinctly different from species to species, resulting in nanoparticles with diverse morphologies [22]. Therefore, it is essential to optimise parameters and to develop controlled procedures to synthesise stable and monodisperse nanoparticles.

AgNP research has grown rapidly over the years due to the optical, antimicrobial and anticancer properties of AgNPs, which make them extremely useful in the biomedicine and biotechnology field [4,5,6,7,23,24]. For example, the inhibitory growth effects of AgNPs have been reported against bacteria such as *Pseudomonas aeruginosa*, *Escherichia coli* (*E. coli*) and *Staphylococcus aureus* (*S. aureus*) [25,26,27]. Furthermore, AgNPs’ anticancer properties were also demonstrated by their cytotoxic effect on human liver cancer cells (HepG2), breast cancer cells (MCF-7) and colorectal cancer cells (HCT116) [28,29,30]. In this scenario, the size of the nanoparticle plays an essential role in its application, as the enhanced antibacterial and anticancer properties were correlated with smaller nanoparticle sizes [25,26,27,31]. Nevertheless, the biological nanoparticle synthesis’s optimum reaction parameters are still not fully elucidated. Therefore, the purpose of this study was (1) to investigate the ability of metabolites secreted by six fungi to synthesise AgNPs and (2) to evaluate the effects of time, temperature and pH variation in combination on the synthesis of AgNPs. This study demonstrated that not all fungi can produce metabolites that can reduce AgNO_3_ into AgNPs. In addition, physicochemical parameters have a significant influence on the stability, size and dispersity of AgNPs. These results highlight that fungal screening is important to determine metabolite functionality. The results also determined optimal parameters for nanoparticle product development.

## 2. Materials and Methods

### 2.1. Microorganisms

The six microorganisms utilised in this were study obtained from the Technological University of Shannon: Midland Midwest (TUS) biobank: *C. bantiana*, *P. antarcticum* (*Centraal Bureau voor Schimmelcultures* CBS 100491), *T. versicolor*, *T. martiale*, *U. isabellina* and *B. adusta*.

### 2.2. Production of Fungal Cell-Free (FCF) Extract

The production of the FCF extract was adapted from AbdelRahim et al., Hamedi et al. and Katapodis et al. [32,33,34]. Fungi were cultivated at room temperature in an MYGP agar medium composed of agar (15 g/L, Formedium, Hunstanton, UK), malt extract (3 g/L, OXOID, Hampshire, UK), yeast extract (3 g/L, Formedium), glucose (10 g/L, Formedium) and peptone (5 g/L Formedium). In aseptic conditions, fungal cells taken from the mycelia mat borders to guarantee active growth were inoculated in 250 mL Erlenmeyer flasks with MYGP medium (previous composition without agar) and placed in an incubator shaker at room temperature at 120 rpm for five days. Cells were washed thoroughly with sterile deionised water, filtered, dried and then accurately weighed. To induce the secretion of secondary metabolites, the cells were transferred to a stress medium, composed of sterile deionised water, with a ratio of 1 g of cells to 10 mL and incubated in the shaker for three days. The fungal secreted extract was separated from the fungus cells by filtration, followed by centrifugation at 5000 rpm for 20 min, at 25 °C, with subsequent 0.22 µm membrane filtration. FCF extracts were stored at 4 °C until further use.

Prior to use, all media were autoclaved at a temperature of 121 °C and pressure of 15 psi for 15 min.

### 2.3. Biological Synthesis of AgNPs

The activity level of the fungal cell-free (FCF) extracts in the synthesis of AgNPs was tested based on the studies of Al-Khuzai et al. [19]. AgNO_3_ at a concentration of 0.5 mM (AgNO_3_, Sigma-Aldrich St. Louis, MO, USA) was used, varying the reaction time (1, 3 and 6 h), temperature (20, 45 and 90 °C) and pH (6, 9 and 12), in the water bath (Julabo TW12, Seelbach, Germany). All reactions were conducted in triplicate.

The pH of the FCF extracts was adjusted using a pH meter (Hach, Cork, Ireland), sodium hydroxide (NaOH) and hydrochloric acid (HCl), both chemicals bought from Sigma-Aldrich (St. Louis, MO, USA). Ultrapure water and AgNO_3_ were used as synthesis reaction control in the same reaction conditions.

### 2.4. AgNPs Characterisation

AgNPs were first characterised by ultraviolet–visible spectrophotometry (UV-Vis, BioTek Synergy 4 Microplate Reader, Bad Friedrichshall, Germany) through localised surface plasmon resonance (LSPR) absorbance measurement, scanned between 300 nm and 1000 nm, with 2 nm steps. Selected AgNPs were then characterised using high-resolution TEM (FEI Titan 80) to evaluate the shape and size distribution of the resulting AgNPs. Samples were drop-coated onto the Formvar carbon-coated copper grids with a 200 µm mesh size (Agar Scientific, Essex, UK) and dried over 24 h. Images were obtained using the FEI Titan 80 operated at 300 kV using a field emission electron gun equipped with a Gatan Ultrascan digital camera. ImageJ software was utilised to measure an average of 200 NPs to evaluate size distribution. Finally, energy-dispersive X-ray spectrometry (EDS) was used to analyse the elemental composition of the synthesised NPs. For this method, 5 µL of the samples was drop-coated trice onto polished aluminium slides and dried in the oven at 60 °C for 1 h. An electron microscope (Hitachi 3000, Tokyo, Japan) at 15 kV accelerating voltage and a working distance of 2 mm was used to obtain the EDS spectra of the samples.

### 2.5. Statistical Analysis

All assays were set in triplicate. Data were expressed as the mean ± standard deviation (St. Dev). The software packages used to analyse the data generated in the characterisation process were Gen5, Microsoft Office Excel and Quantax 70 Microanalysis.

## 3. Results

### 3.1. Production of Fungal Cell-Free Extract

**Figure 1:**
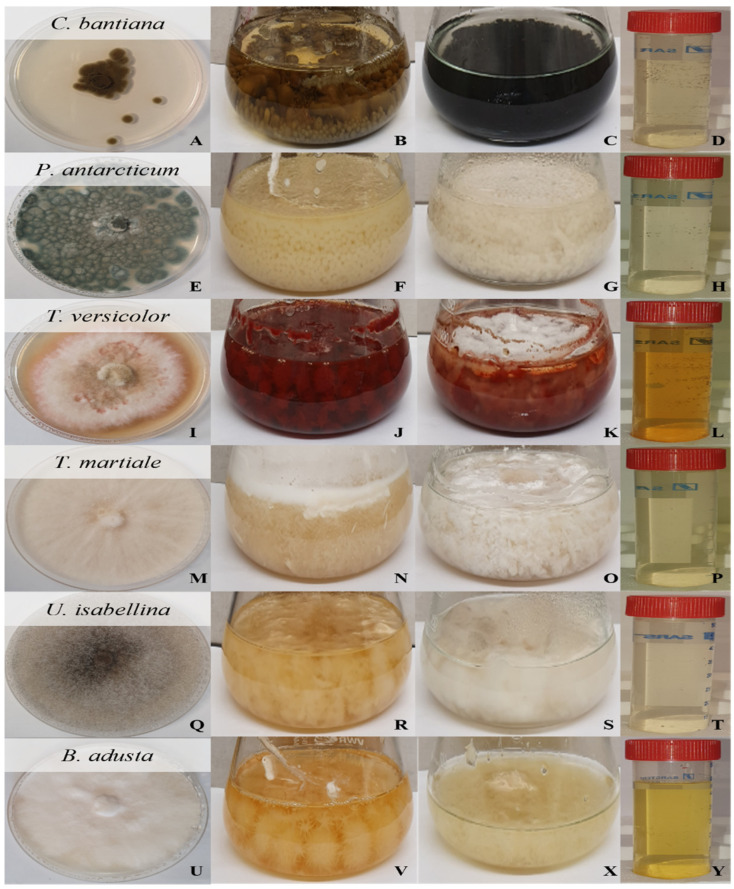
Fungus growth in MYPG agar plates (first column), MYGP medium (second column), stress medium (third column) and the fungal cell-free extract (fourth column): *Cladophialophora bantiana* (**A**–**D**), *Penicillium antarcticum* (**E**–**H**), *Trametes versicolor* (**I**–**L**), *Trichoderma martiale* (**M**–**P**), *Umbelopsis isabellina* (**Q**–**T**) and *Bjerkandera adusta* (**U**–**Y**).

Fungi were initially cultured on MYPG agar plates and are displayed in Figure 1.

The mycelia weight of each fungus after fungal growth in MYGP medium and the pH of the FCF extract after the growth phase (MYGP medium) and after the stress phase (sterile deionised water) are displayed in Table 1. *P. antarcticum* was revealed to have the fastest growth in MYGP medium, which correlates with greater volume production of the FCF stress extract that can lead to future scale-up evaluation. An increase in the pH of the extracts, from growth to stress phase, was observed for the majority of the fungi except for the *U. isabellina* FCF extract, which exhibited no change in the pH between the two phases.

### 3.2. Biological Synthesis of AgNPs

The activity level of the FCF stress extracts was tested by the addition of 0.5 mM AgNO_3_, varying the reaction time (1, 3 and 6 h), temperature (20, 45 and 90 °C) and pH (6, 9 and 12). All reactions were conducted in triplicate. The presence of a clear and defined LSPR band in the region of 400 nm in the UV-Vis spectrophotometric analysis indicates AgNP synthesis.

**Figure 2:**
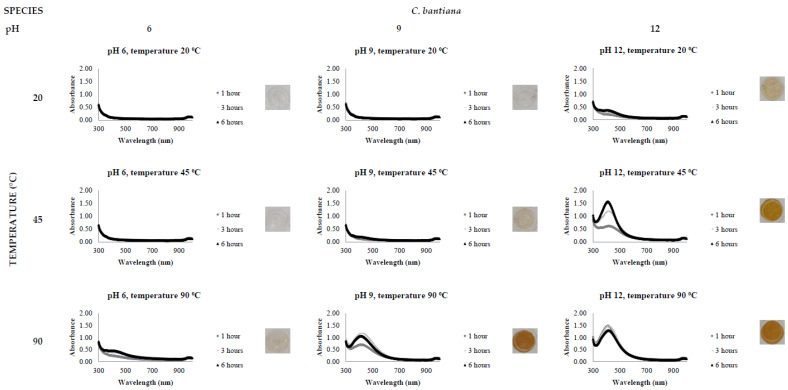
LSPR spectra of *C. bantiana* cell-free extract in the synthesis of AgNPs, by the addition of 0.5 mM AgNO_3_, varying the reaction time (1, 3 and 6 h), temperature (20, 45 and 90 °C) and pH (6, 9 and 12), through UV-Vis spectrophotometry analysis.

**Figure 3:**
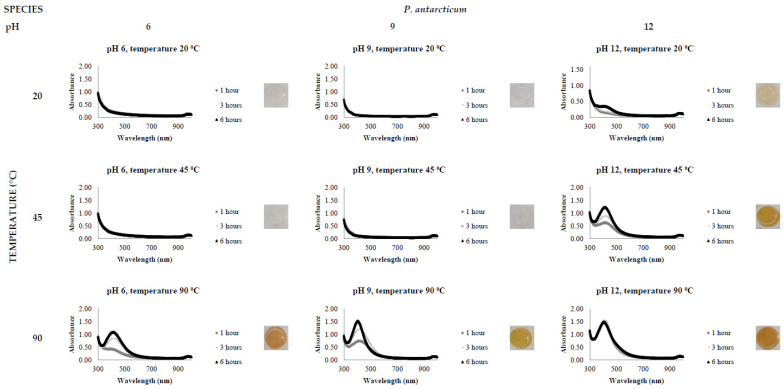
LSPR spectra of *P. antarcticum* cell-free extract in the synthesis of AgNPs, by the addition of 0.5 mM AgNO_3_, varying the reaction time (1, 3 and 6 h), temperature (20, 45 and 90 °C) and pH (6, 9 and 12), through UV-Vis spectrophotometry analysis.

**Figure 4:**
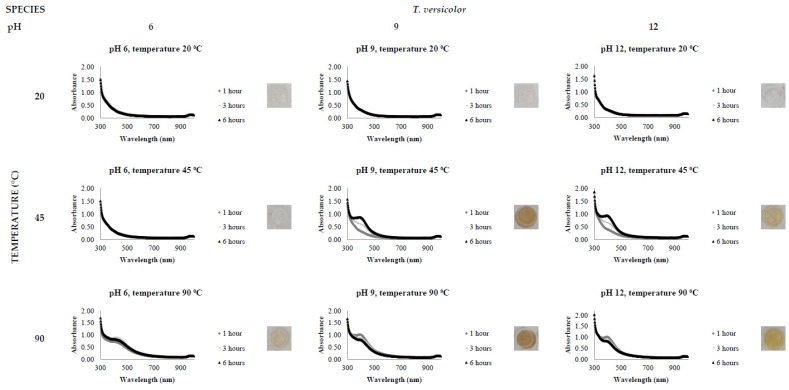
LSPR spectra of *T. versicolor* cell-free extract in the synthesis of AgNPs, by the addition of 0.5 mM AgNO_3_, varying the reaction time (1, 3 and 6 h), temperature (20, 45 and 90 °C) and pH (6, 9 and 12), through UV-Vis spectrophotometry analysis.

**Figure 5:**
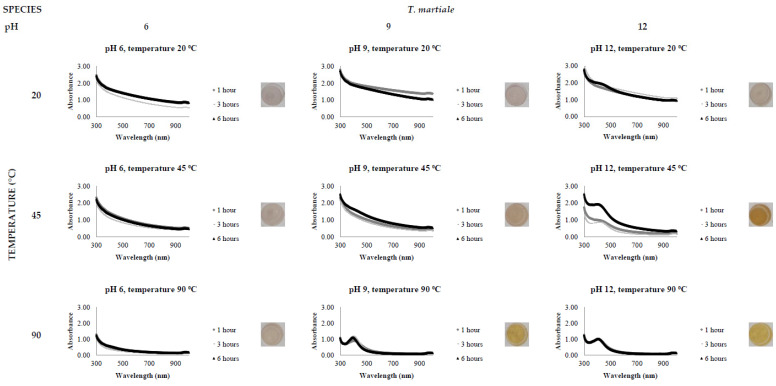
LSPR spectra of *T. martiale* cell-free extract in the synthesis of AgNPs, by the addition of 0.5 mM AgNO_3_, varying the reaction time (1, 3 and 6 h), temperature (20, 45 and 90 °C) and pH (6, 9 and 12), through UV-Vis spectrophotometry analysis.

**Figure 6:**
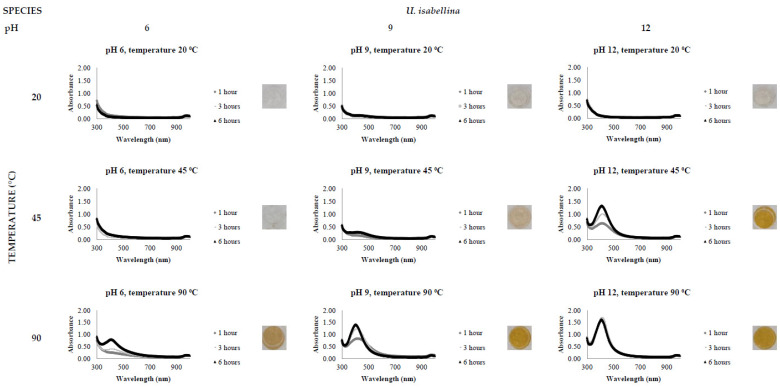
LSPR spectra of *U. isabellina* cell-free extract in the synthesis of AgNPs, by the addition of 0.5 mM AgNO_3_, varying the reaction time (1, 3 and 6 h), temperature (20, 45 and 90 °C) and pH (6, 9 and 12), through UV-Vis spectrophotometry analysis.

**Figure 7:**
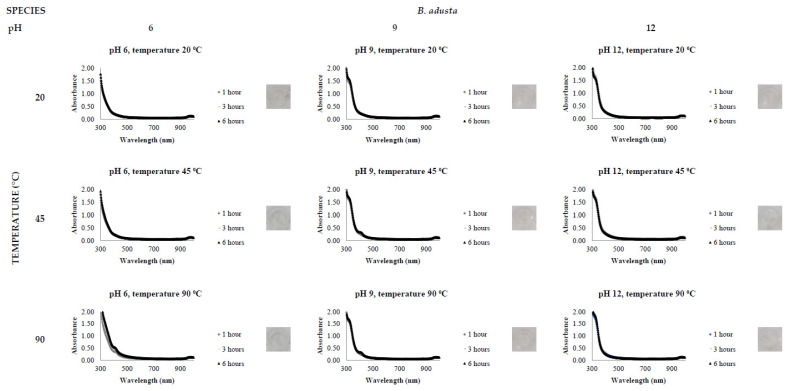
LSPR spectra of *B. adusta* cell-free extract in the synthesis of AgNPs, by the addition of 0.5 mM AgNO_3_, varying the reaction time (1, 3 and 6 h), temperature (20, 45 and 90 °C) and pH (6, 9 and 12), through UV-Vis spectrophotometry analysis.

Figure 2, Figure 3, Figure 4, Figure 5, Figure 6 and Figure 7, respectively, show the UV-Vis spectrum results of the 27 reactions in the following order: *C. bantiana*, *P. antarcticum*, *T. versicolor*, *T. martiale*, *U. isabellina* and *B. adusta* (Figure 2, Figure 3, Figure 4, Figure 5, Figure 6 and Figure 7). *P. antarticum* and *T. versicolor* FCF stress extracts were the only ones capable of synthesising AgNPs at all the pH values tested with a defined LSPR band in the region of 400 nm (Figure 3 and Figure 4). On the other hand, *B. adusta* FCF stress extract could not synthesise AgNPs in any conditions analysed (Figure 7).

It was possible to notice that higher temperatures associated with a basic pH led to a quicker reaction time, a narrower spectrum (indicative of a monodisperse nanoparticle size distribution) and higher absorbance values (indicative of a greater AgNO_3_ to AgNP conversion rate). In addition, it was shown that the pH significantly affects AgNP synthesis at higher temperatures, which can be visually observed by examining the LSPR absorbance spectrum. Therefore, the reaction conditions of 0.5 mM AgNO_3_, 90 °C, different pH values (6, 9 and 12) and one hour reaction time were selected for UV-Vis spectrophotometric statistical analysis (Table 2).

### 3.3. AgNP Characterisation

**Figure 8:**
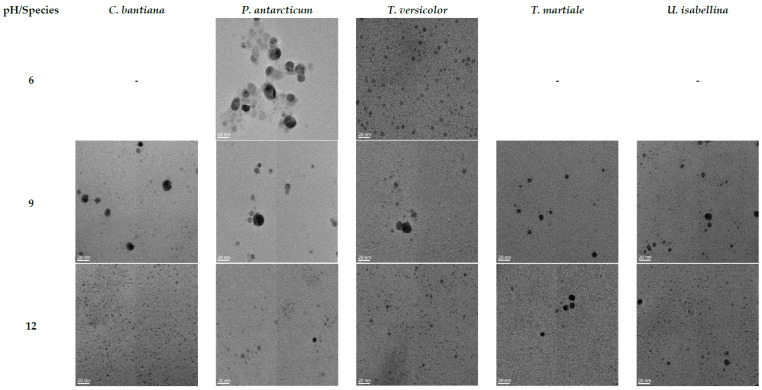
TEM (20 nm scale) characterisation of the biological AgNPs. AgNPs were synthesised by adding 0.5 mM AgNO_3_, with 1 h reaction time, at a temperature of 90 °C, varying the pH. Images of the AgNPs synthesised at pH 6 are shown in the second row, pH 9 in the third row and pH 12 in the last row. AgNPs synthesised using the fungal cell-free extracts of *C. bantiana* are shown in the second column, *P. antarcticum* in the third column, *T. versicolor* in the fourth column, *T. martiale* in the fifth column and *U. isabellina* in the last column.

The AgNPs synthesised using fungal stress extract and 0.5 mM AgNO_3_ at 90 °C and different pH values (6, 9 and 12) for one hour reaction time were selected for further TEM characterisation and size distribution analysis, as shown in Figure 8. TEM images, on a 20 nm scale, indicated that spherical-shaped AgNPs were synthesised. EDS analysis (data not shown) was carried out on the samples, and the presence of elemental silver was confirmed at 3 keV where AgNPs were identified in TEM.

Furthermore, it was possible to note that smaller nanoparticles were synthesised when changing the pH from acid to basic. In addition, the percentage of the nanoparticles in the same size range increased, decreasing the polydispersity of the AgNPs solutions. The statistical analysis regarding the AgNPs’ size distribution is shown in Table 3.

## 4. Discussion

Research into AgNP synthesis using eco-friendly approaches has grown rapidly because of the promising applications of these nanoparticles within the biomedicine and biotechnology field. In this context, myco-synthesis has been investigated thoroughly due to the tolerance and metal bioaccumulation capacity of fungi. Other reasons include economic viability, ease in handling biomass during downstream processing and large-scale production, which are advantages of using fungi rather than other microorganisms [14].

Fungi species such as *Aspergillus fumigatus*, *Cladosporium halotolerans*, *Fusarium oxysporum* and *Trichoderma longibrachiatum* have been previously shown to synthesise AgNPs [35,36,37,38]. However, despite efforts to establish controlled procedures to synthesise AgNPs biologically, the optimum reaction parameters display contradicting results and variability. Fungi are living organisms with different capabilities; therefore, optimum parameters for one species can be completely different from those for another. This could be due to the difference in the metabolic pathway response under stress conditions and the biosynthesis mechanism for each organism [19].

Several studies explore the effect of one parameter individually instead of analysing the combined impact of multiple variables [21,22,39,40]. Therefore, this study explored the ability of six different fungi, *C. bantiana*, *P. antarcticum*, *T. versicolor*, *T. martiale*, *U. isabellina* and *B. adusta*, to synthesise AgNPs, evaluating the combined effect of time, temperature and pH.

The results of this study demonstrate the following: (1) Nanoparticles produced by the same fungi metabolites can vary in both size and dispersion. This variance is correlated with physiochemical reaction parameters. (2) Optimal conditions to synthesise stable, monodisperse and smaller NPs were found at a higher pH of 12 and at a higher temperature of 90 °C. (3) Not all fungi (*B. adusta*) could reduce AgNO_3_ precursors into AgNPs, demonstrating the importance of fungal screening studies.

The influence of pH (3 to 11) on the biological synthesis of AgNPs has been previously explored [19,20,21]. Studies have shown that basic pH is optimal for biological AgNP synthesis. For example, studies using extracts of the fungi *Saprolegnia parasitica*, *Neurospora crassa* and *Sclerotinia sclerotiorum* demonstrated alkali conditions as optimum in the myco-synthesis of AgNPs [19,20,21]. Our UV-Vis spectrophotometer study (Figure 2, Figure 3, Figure 4, Figure 5, Figure 6 and Figure 7) demonstrated that FCF extracts of only two out of the six species (*P. antarcticum* and *T. versicolor*) were capable of reducing AgNO_3_ into AgNPs in acidic pH (6) at an elevated temperature (90 °C). This may be caused by the formation of silver chloride (AgCl) when HCl is used to change the pH of the solution, which prevents the formation of AgNPs. Furthermore, it was also shown that basic pH (9 and 12) associated with high temperatures (45 and 90 °C) led to a quicker reaction time, a higher absorbance yield and a narrower spectrum, indicative of a monodisperse nanoparticle size distribution. Our findings could be explained by the fact that basic pH benefits the reduction of Ag+ ions to produce AgNPs by providing electrons for the reaction. As a result, more hydroxide (OH-) anions are available to participate in the reduction reaction, increasing the reduction strength [23,33].

The influence of temperature on the biological synthesis of nanoparticles is a cause for contradicting results. However, the results obtained in this research are similar to those found by Saxena et al., who reported maximum synthesis achieved at the highest temperature studied [21], and by Al-Khuzai et al., who reported a reduction in the nanoparticle size when increasing the temperature from 25 to 90 °C [19]. Previous biological AgNP synthesis studies analysed the reaction between 3 and 48 h [22,32]. Our UV-Vis spectrophotometer study (Figure 2, Figure 3, Figure 4, Figure 5, Figure 6 and Figure 7) demonstrated that AgNPs completed synthesis within the first hour at basic pH and elevated temperature (90 °C). In this context, it is essential to notice that for rapid synthesis, the reaction rate increases with the increase in the temperature. However, the denaturation or inactivation of potential enzymes and other active molecules responsible for the synthesis can also occur at elevated or low temperatures [8,32]. As higher temperatures were used for synthesis, reducing and stabilising capacity was not correlated with enzyme activity as denaturation occurs at this temperature.

It is known that the composition, shape and size of the nanoparticles determine their physical and chemical properties, such as reactivity, biological interactions and optical properties [41,42]. Therefore, establishing an accurate relationship between the reaction parameter conditions and their effect on the morphology is extremely important for their future efficacy and performance applications. Several studies demonstrated the influence of the size and shape on the AgNPs’ properties, revealing enhanced antibacterial and anticancer properties correlated with smaller nanoparticle sizes [25,26,27,28,29,30]. For example, the antibacterial effect of AgNPs in the size range of 5 to 100 nm was tested against *E. coli*, *Bacillus subtilis* and *S. aureus*, showing the fastest bactericidal activity with the use of the smallest nanoparticle size [25]. Moreover, it was demonstrated that chemically made small spherical-shaped AgNPs had enhanced antibacterial activity against *Pseudomonas aeruginosa* and *E. coli* compared to larger ones and also compared to triangular-shaped AgNPs [26]. The microscopy study revealed not only that we established defined parameters to synthesise nanoparticles of different sizes using the same fungal cell-free stress extract but also demonstrated that at a high temperature, when increasing the reaction pH, smaller nanoparticles were synthesised and the percentage of the nanoparticles in the same size range increased, hence positively increasing the monodispersity of the AgNP solutions (Figure 8 and Table 3). Table 3 displays the sizes of the AgNPs synthesised using 0.5 mM AgNO_3_ for 1 h reaction time at 90 °C and basic pH (9 and 12), which were less than 10 nm. Furthermore, Figure 8 displays spherical-shaped AgNPs. Therefore, the biologically synthesised AgNPs shown in this study have potential as sustainable substitutes for chemically made antibacterial and anticancer products. Hence, they represent a step in the right direction towards achieving a bio-based circular economy.

## 5. Conclusions

As sustainability concerns grow, there is a shift in focus to a bio-based circular economy. Fungi have the ability to convert organic materials into bioactive products in an environmentally friendly manner. AgNPs have huge potential in biomedicine and biotechnology applications, especially due to their enhanced antibacterial and anticancer properties. However, chemically made AgNPs can be environmentally harsh. On the other hand, fungi biological synthesis, as we describe, can be used as a sustainable route to greener NP synthesis. However, the biological synthesis mechanism is still not fully established. Hence, this work evaluated the combined effect of pH, time and temperature in the mycosynthesis of AgNPs. Five fungi out of six tested had the ability to produce metabolites that can synthesise spherical AgNPs, albeit with different sizes (3 to 17 nm) and dispersity percentages (25 to 95%, within the same size range). We conclude that smaller and monodisperse AgNPs were favourably synthesised at elevated temperatures (90 °C) associated with basic pH (9 and 12).

## Figures and Tables

**Table 1:** Mycelia weight and FCF extract pH of each fungus in the growth phase (MYGP medium) and FCF extract pH after the stress phase (sterile deionised water).

Species	Growth Phase		Stress Phase
	Mycelia (g)	FCF Extract pH	FCF Extract pH
*C. bantiana*	7.0	5.4	7.9
*P. antarcticum*	10.4	6.5	7.1
*T. versicolor*	9.3	7.2	7.9
*T. martiale*	8.8	5.2	7.7
*U. isabellina*	7.4	7.8	7.8
*B. adusta*	4.5	5.3	8.3

**Table 2:** UV-Vis spectrophotometric statistical analysis of the wavelength associated with a maximum absorbance of the AgNPs synthesised using FCF extract, 0.5 mM AgNO_3_, 1 h reaction time, 90 °C and different pH values (6, 9 and 12).

Species	AgNPs		
	pH	Wavelength (nm) Mean ± St. Dev	Absorbance Mean ± St. Dev
*C. bantiana*	6	Nd ^1^	Nd ^1^
	9	413 ± 1.018	0.706 ± 0.013
	12	411 ± 1.018	1.479 ± 0.019
*P. antarcticum*	6	390 ± 2.828	0.449 ± 0.013
	9	415 ± 1.414	0.746 ± 0.020
	12	410 ± 0.000	1.523 ± 0.028
*T. versicolor*	6	390 ± 4.000	0.729 ± 0.036
	9	392 ± 0.000	1.019 ± 0.018
	12	394 ± 0.000	1.016 ± 0.046
T. martiale	6	Nd ^1^	Nd ^1^
	9	404 ± 0.000	0.892 ± 0.028
	12	405 ± 1.414	1.010 ± 0.029
*U. isabellina*	6	Nd ^1^	Nd ^1^
	9	415 ± 1.155	0.833 ± 0.018
	12	410 ± 0.000	1.599 ± 0.087
*B. adusta*	6	Nd ^1^	Nd ^1^
	9	Nd ^1^	Nd ^1^
	12	Nd ^1^	Nd ^1^

^1^ Nd, not determined.

**Table 3:** Size statistical analysis of the AgNPs synthesised using FCF extract, 0.5 mM AgNO_3_, 1 h reaction time, 90 °C and different pH values (6, 9 and 12), calculated using ImageJ.

Species	pH	AgNP Size (nm) Mean ± St. Dev	Highest Percentage within the Size Range
*C. bantiana*	6	Nm ^1^	Nm ^1^
	9	7.019 ± 4.494	41.0% (4–7.99 nm)
	12	3.062 ± 1.423	94.5% (0–3.99 nm)
*P. antarcticum*	6	16.811 ± 8.580	25.0% (16–19.99 nm)
	9	7.884 ± 4.183	50.5% (4–7.99 nm)
	12	5.943 ± 2.364	76.5% (4–7.99 nm)
*T. versicolor*	6	6.526 ± 1.459	78.0% (4–7.99 nm)
	9	7.336 ± 6.707	48.0% (0–3.99 nm)
	12	4.816 ± 3.503	65.0% (0–3.99 nm)
*T. martiale*	6	Nm ^1^	Nm ^1^
	9	3.214 ± 2.654	70.0% (0–3.99 nm)
	12	4.051 ± 2.640	86.5% (0–3.99 nm)
*U. isabellina*	6	Nm ^1^	Nm ^1^
	9	4.239 ± 2.920	67.5% (0–3.99 nm)
	12	3.676 ± 1.818	78.5% (0–3.99 nm)
*B. adusta*	6	Nm^1^	Nm ^1^
	9	Nm^1^	Nm ^1^
	12	Nm^1^	Nm ^1^

^1^ Nm, not measured.

## Data Availability

Not applicable.
